# Environmental Enrichment Increases Brain Volume in Snakes

**DOI:** 10.1002/cne.70043

**Published:** 2025-03-21

**Authors:** Gokulan Nagabaskaran, Vijay Moonilal, Morgan Skinner, Noam Miller

**Affiliations:** ^1^ Department of Psychology Wilfrid Laurier University Waterloo Canada

**Keywords:** 3D Slicer, brain, environmental enrichment, MRI, reptile welfare, reptile, RStudio, snake

## Abstract

The effects of environmental enrichment have been well documented in mammals and birds, but less work has focused on reptiles. Because snakes are common in captivity, both as pets and in research/commercial facilities, it is critical to explore how they react to standard captive housing. Here, we examined the effects of environmental enrichment on brain development in a popular pet snake species, the western hognose snake (*Heterodon nasicus*). Hognose snakes (*n* = 15) were individually housed for one year in either enriched or standard environments before their brains were harvested and imaged using MRI. We found that enriched snakes had significantly larger brain volumes compared to standard snakes, most prominently in posterior brain regions. In addition, we observed sex‐specific brain investments: as snakes grew larger, males displayed relatively larger cerebral hemispheres, and females displayed larger posterior brain regions. These results suggest that environmental enrichment is critical to encouraging healthy brain development in snakes and that snake brain plasticity is very similar to that observed in mammals and birds.

## Graphical abstract

MRI scans of Western hognose (*Heterodon nasicus*) brains show that snakes that lived in enriched conditions for 8 months have larger brains than snakes that lived in impoverished conditions, especially in the hindbrain. Relative brain area sizes differed across sexes.

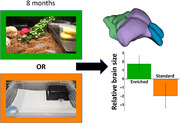

## 1 Introduction

Many animals in captivity are housed under less than ideal conditions. Captive environments may lack critical aspects of a species’ natural habitat and may not allow animals to engage in natural behaviors important to their health and well‐being (Young et al. 2020). Deficiencies in captive environments can be identified by improvements in cognition and health—as well as behavioral changes—that emerge when environments are improved or enriched (Olsson and Dahlborn 2002; Tahamtani et al. 2018; Toli et al. 2017). Environmental enrichment (EE) can be defined as changing a captive animal's environment in a beneficial manner that encourages natural behaviors (Widenmayer 1996; Young et al. 2020), which may reduce stress and improve health (Leal‐Galicia et al. 2008; Coulton et al. 1997). EE can take many forms and should be tailored to the ecology of the animal (Young et al. 2020). One common method of EE is to increase the complexity of the captive environment using physical structures (Depasquale et al. 2016) that allow animals to engage in natural behaviors such as burrowing, nesting, or climbing (Van de Weerd et al. 1996; Hoehfurtner et al. 2021).

EE can lead to cognitive benefits, such as improved spatial learning, increased exploration, increased memory retention, and decreased anxiety (Jones and Waddington 1992; Leal‐Galicia et al. 2008; Depasquale et al. 2016). Such cognitive changes are inevitably linked to changes in the brain, with studies observing increases in brain size (Cummins et al. 1973; Scotto Lomassese et al. 2000), increased neurogenesis (Depasquale et al. 2016; Leal‐Galicia et al. 2008; Segovia et al. 2006), and effects on disease progression (Wolf et al. 2006). Increases in brain size as a result of enrichment have been observed in various animal models and are sometimes age‐dependent (Cummins et al. 1973; Fong et al. 2019; Näslund et al. 2012).

In fish, enrichment primarily affects growth in the hippocampal formation (Depasquale et al. 2016), but inconsistent results have been found in other taxa. When stones were added as enrichment to juvenile steelhead salmon tanks (*Oncorhynchus mykiss*), their cerebellums grew larger compared to fish in barren tanks (Kihslinger and Nevitt 2006). However, coho salmon (*Oncorhynchus kisutch*) reared in simplistic hatchery environments grew larger brains overall than fish raised in a complex stream‐like environment (Kotrschal et al. 2012). Other studies have also produced mixed, little, or no effects as a result of enrichment across various species (Toli et al. 2017; Depasquale et al. 2016; Kihslinger and Nevitt 2006).

Little work has been done on EE in reptiles, compared to mammals and birds, even though reptiles engage in complex behaviors that include social communication, social learning, parental care, and play (Font et al. 2023). The few reptilian studies conducted have been inconclusive, with some showing a positive effect of enrichment and others not, depending on factors such as ontogeny and species‐specific needs (Burghardt and Layne 1995; Nagabaskaran 2020). Rat snakes (*Elaphe obsoleta*) exposed to EE displayed quicker habituation rates and performed better in goal‐oriented tasks compared to unenriched snakes (Almli and Burghardt 2006). Enriched corn snakes (*Pantherophis guttatus*) displayed discrimination of a familiar handler based on odor cues and familiar objects that lacked odor cues, while snakes housed without enrichment did not (Nagabaskaran et al. 2021; Nagabaskaran 2020). Ball pythons (*Python regius*) and Madagascar giant hognose snakes (*Leioheterodon madagascariensis*) display significantly fewer stress‐induced abnormal behaviors in enriched environments (Hollandt et al. 2021; Spain et al. 2020), and leopard geckos (*Eublepharis macularius*) display high levels of interest and motivation for enrichment after being exposed to barren environments (Zielikski 2023). Moreover, both western hognose snakes (*Heterodon nasicus*) and corn snakes show a clear preference for EE when given a choice between enriched and unenriched environments (Nagabaskaran et al. 2022; Hoehfurtner et al. 2021). These studies used naturalistic EE that allowed for climbing, burrowing, and access to multiple shelters that varied in elevation and humidity. Such components of enrichment, which reflect the species’ natural environment, should be used widely with all captive reptiles to improve reptilian health and welfare (Warwick and Steedman 2023), as a survey of 675 snakes worldwide indicated that snakes with larger enclosures and enrichment stimuli displayed fewer abnormal clinical signs (Cargill et al. 2022).

In contrast, a few studies have found no significant effects of enrichment on reptiles. For example, arboreal fence lizards (*Sceloporus undulatus)* showed no change in either natural behaviors or stress hormone levels when provided with a 14 cm high climbing enrichment (Rosier and Langkilde 2011), which may have simply been inadequate given the much larger heights these lizards naturally climb to (Kennedy 1958). Similarly, green anoles (*Anolis carolinensis*) and corn snakes displayed no significant changes in preference, behavior, or physiological markers when exposed to EE (Borgmans et al. 2018; Evans 2011). In addition, box turtles (*Terrapene carolina*) displayed some significant changes when exposed to enrichment, but these effects were transient across multiple timepoints (Tetzlaff et al. 2019). Given this inconsistency in the data on enrichment in reptiles, further investigation is clearly required.

Even less work has been done on the effects of enrichment on the brains of reptiles. A territorial morph of side‐blotched lizards (*Uta stansburiana*) displayed neurogenesis when given EE in the form of a larger enclosure, though non‐territorial morphs did not (LaDage et al. 2013), and there is a mention of enriched monitor lizards displaying a trend for larger forebrains in an unpublished note (in Burghardt 2013). These results hint that reptilian brains are plastic, like those of other taxa, and that they may be affected by environmental factors.

**FIGURE 1:**
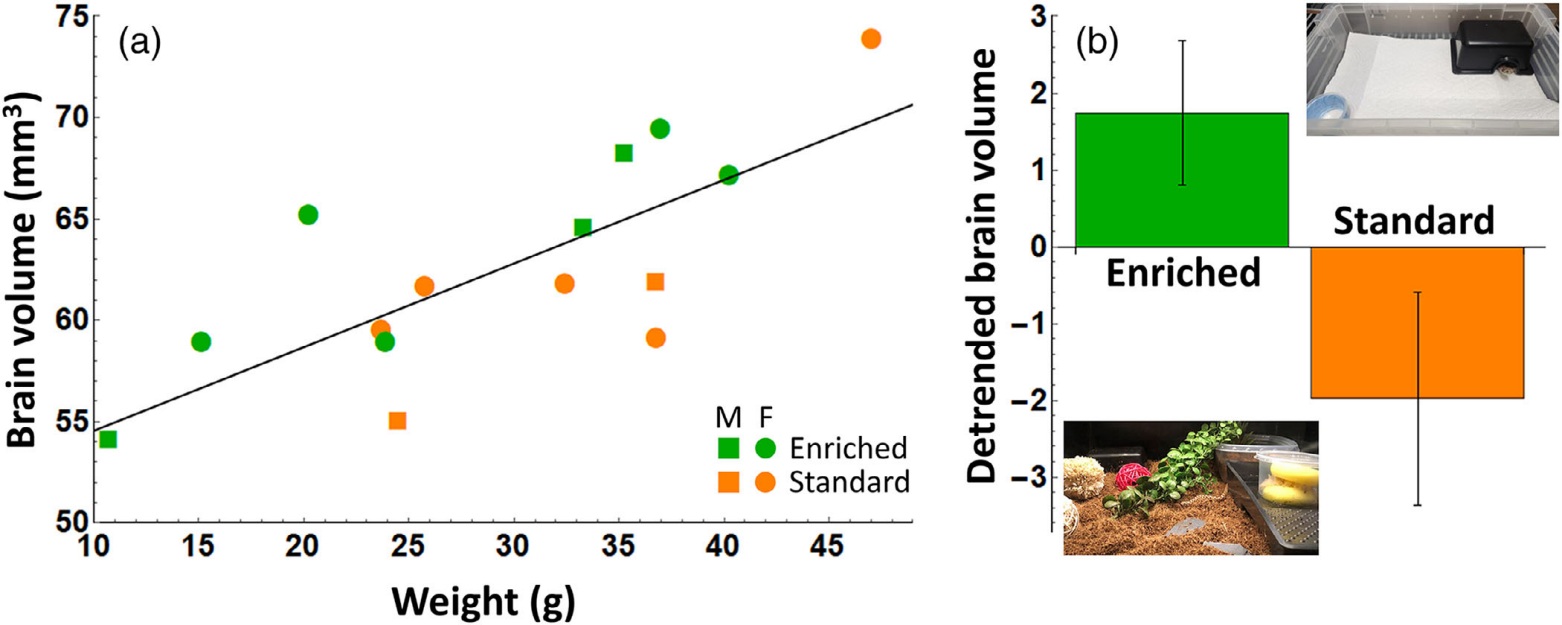
Brain volume as a function of weight, sex, and enrichment. (a) Brain volume in mm^3^ as a function of snake weight in g for male (M, square) and female (F, circle) snakes in the enriched (green) and standard (orange) conditions. The black diagonal line shows a linear regression on the data. (b) Brain volumes for the enriched (green) and standard (orange) snakes, controlled for snake weight; error bars show ± SEM. The insets show photographs of the enriched and standard environments.

To better understand the effects of enrichment on reptile brains, we assessed whether and how providing EE affected brain volume in juvenile western hognose snakes. This species is semi‐fossorial and is known for its unique shovel‐like nose that it uses to dig underground shelters. They are opportunistic feeders that prey on both aquatic and terrestrial organisms (Averill‐Murray 2006). We exposed half our snakes to species‐specific enrichment and housed the remaining subjects in a relatively barren rack system, similar to those commonly used by breeders and pet owners (Figure 1b). After living in their respective treatments for approximately one year, we obtained MRI scans of all the snakes’ brains. Paralleling findings in other taxa, we hypothesized that exposure to EE would increase brain volumes in developing snakes.

## 2 Methods

### 2.1 Animals and Husbandry

A total of 15 captive‐bred western hognose snakes (five males) were acquired from a local breeder. The snakes weighed an average of 4.3 ± 3.2 g at the start of the study and were 2–3 months old. They were housed individually in either enriched or standard enclosures (see below) for the duration of the study (∼1 year). The snakes were fed two pieces of human‐grade defrosted salmon (great value) dusted with reptile calcium supplement (Zilla) weekly on the same day, from within individual feeding boxes (11 cm × 15 cm × 4 cm). Each piece of salmon was slightly larger than the snake's head. In both conditions, snakes were given fresh water daily. Any feces or sheds found were removed during daily checks, and when snakes in the standard housing condition defecated, the entire paper‐towel flooring was replaced.

### 2.2 Housing Conditions

Three males and five females were housed in the enriched condition, and the remaining two males and five females were housed in the standard condition. The enriched enclosures (46 cm × 56 cm × 30 cm) were constructed of PVC with a sliding glass door (43 cm × 12 cm) in the front. Enrichment was chosen based on preliminary preference testing (Nagabaskaran et al. 2022) and consisted of approximately 5 cm deep loose coconut husk substrate (Zoo Med Eco), a single black plastic shelter (Cornel's World; 14 cm × 10 cm × 5 cm), a large water dish (27 cm × 15 cm × 6 cm), a sand dish for burrowing, plastic vine (Cornel's World), a damp shelter (10 cm × 7 cm), and straw balls (5 cm diameter) for rooting behavior (see Figure 1b). Enclosures included LED lighting (at 2700 K) on the ceiling with a 12:12 h cycle (lights on at 8:30 a.m.), and heat was provided via thermostat‐controlled heat tape (THGHeat; Spyder Electronics HerpStat) under one corner of the enclosure. This created a heat gradient within the enclosure ranging from 23°C to 32°C. Heat tape was used as it maintains constant temperatures better than heat lamps and mimics commonly used heating setups by pet owners.

Snakes in the standard condition were housed in drawer‐sized boxes (24 cm × 38 cm × 8 cm) placed within bare enclosures similar in size to the enriched enclosure. The same light cycle and heating were provided. The enclosures had paper towel sheets as bedding, a single black shelter (Cornel's World; 14 cm × 10 cm × 5 cm), and a small water dish in the form of a plastic sauce cup (6 cm × 3 cm).  

### 2.3 Perfusion/MRI Methodology

Snake brains were prepared and scanned using well‐established procedures for imaging small animal brains, for which euthanasia is required (Spring et al. 2007; Cahill et al. 2012; Ellegood et al. 2014). Snakes were anesthetized with an overdose of sodium pentobarbital (100 mg/kg body weight) injected intracardially, based on weights taken immediately prior to sedation. Snakes were checked carefully for reactivity after dosing to ensure proper sedation via strong tail pinches and assessment of muscle tone and only progressed to perfusion after body muscle contractions and tongue movements in reaction to pinching had ceased. They were perfused transcardially with 100 mL of 0.1 M phosphate buffered solution (PBS; pH 7.3) containing 2 mM ProHance (a contrast agent; gadoteridol, Bracco Diagnostics Inc., Princeton, NJ) followed by 100 mL of 4% paraformaldehyde with 2 mM ProHance. This was necessary to allow the contrast agent to perfuse the brain. After perfusion, the snakes were immediately decapitated, and the tissue surrounding the skull was removed. The skulls containing the brains were placed in 4% paraformaldehyde with 2 mM ProHance at 4°C for approximately 12 h immediately after surrounding tissue was removed. The skulls were then transferred to a 0.1 M PBS solution containing 2 mM ProHance and 0.02% sodium azide until they were scanned.

Brain images within the skulls were acquired using a 3 Tesla MRI scanner (Siemens PrismaFit) at The Center for Phenogenomics (TCP) in Toronto, Canada. Images were T2‐weighted. All images were processed using 3D Slicer 5.2.2 (Federov et al. 2012; https://www.slicer.org) by a researcher blind to the identities of the snakes and their housing condition (VM). Segmentation was performed using 3D Slicer's automated *Grow from seeds* tool, combined with manual adjustment when necessary. Complete brains were further segmented into three subsections based on easily identifiable morphological features of the snake brain. This involved segmentation at the junction of the olfactory bulbs (OBs) and the cerebral hemispheres, as well as at the sulcus where the forebrain and midbrain meet (Figure 3a). These three subsections are hereafter referred to as the OBs (including the main OB, accessory OB, and the olfactory tract), the cerebral hemispheres (CH, approximately overlapping with the pallium), and the posterior brain (PB; the midbrain and hindbrain, including the optic tectum, cerebellum, and brainstem). Overall brain volumes (in mm^3^), as well as the volumes of each subsection, were calculated using 3D Slicer's *segment statistics* module. Volumes for each subsection were converted to proportions of total brain volume.

### 2.4 Statistical Analysis

Analyses were performed in R (version 4.2.1, R Core Team, 2022). We modeled the factors important for predicting brain volume by progressively adding weight, sex, a weight by sex interaction, and housing condition to an analysis of variance. We compared the resulting models by their AIC values and dropped factors that did not improve the model fit. A model was considered to be a significantly better fit if it improved the AIC by a value of 2 or more (Cavanaugh and Neath 2019). As brain volumes were strongly correlated with snakes’ overall weight (see Results), we included weight as a covariate in all models.

## 3 Results

**FIGURE 2:**
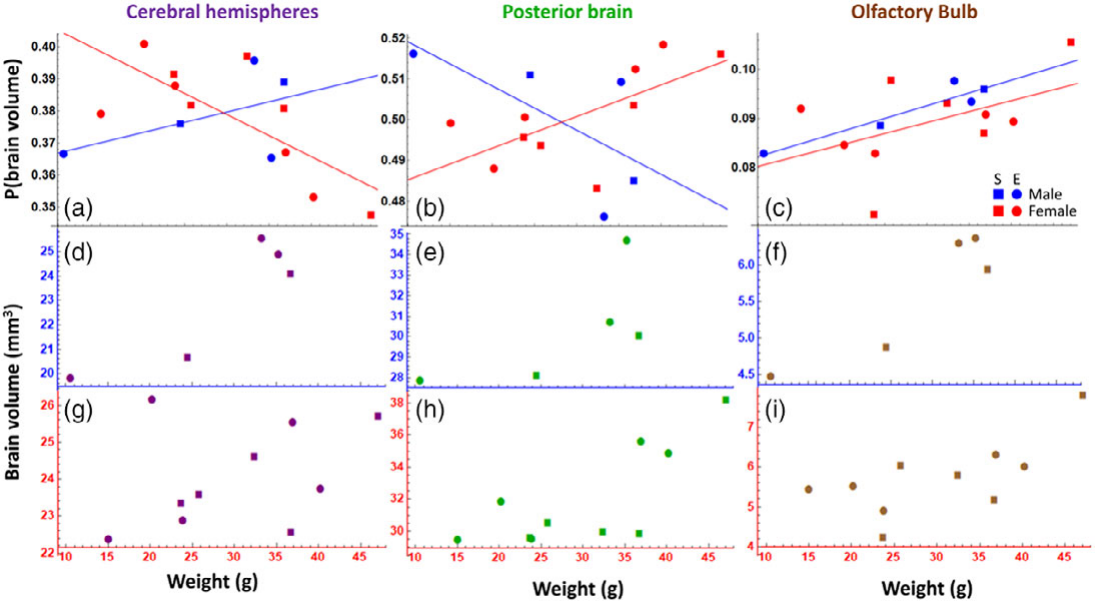
Relative (a–c) and absolute (d–i) brain area volumes as a function of weight, sex, and enrichment. a, d, g: forebrain; b, e, h: mid/hindbrain; c, f, i: olfactory bulb. Panels (a–c) show the proportion of total brain volume consisting of that brain area as a function of snake weight in g for male (blue) and female (red) snakes in the enriched (circle) and standard (square) conditions. The blue and red lines in each panel show linear regressions. Panels (d–f) show absolute brain volumes (in mm^3^) of male snakes in the enriched (circle) and standard (square) conditions, and panels (g–i) show the same data for female snakes.

Larger snakes had larger brain volumes (Figure 1a; *F*(1,12) = 22.54, *p* < 0.001), and snakes in the enriched housing condition had larger brains than controls (Figure 1b; *F*(1,12) = 5.34, *p* = 0.039). Further analysis found that most of the increase in volume for snakes in the enriched condition occurred in the PB (Figure 2e,h; *F*(1,12) = 6.73, *p* = 0.025; enriched mean = 31.81 ± 2.91, standard mean = 30.82 ± 3.31), with no significant effect found in the cerebral hemispheres (Figure 2d,g; *F*(1,12) = 1.76, *p* = 0.209; enriched mean = 23.86 ± 2.12, standard mean = 23.46 ± 1.60) or the OBs (Figure 2f,i; *F*(1,12) = 1.43, *p* = 0.256; enriched mean = 5.66 ± 0.71, standard mean = 5.66 ± 1.14). Larger snakes had larger brains in all three subregions (all *F*’s > 7.00, all *p*’s < 0.05).

**FIGURE 3:**
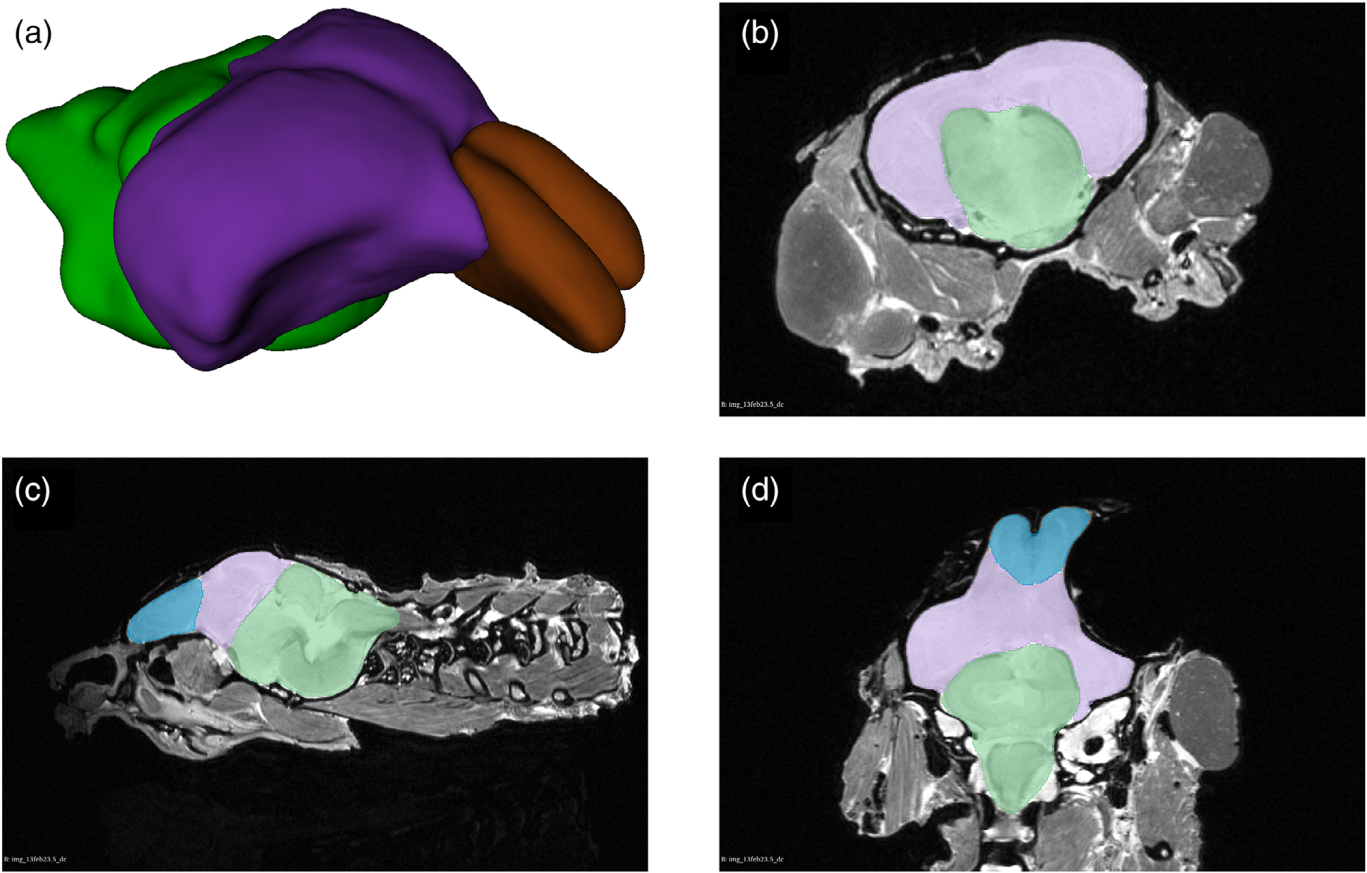
Brain segmentation. (a) 3D view of a sample segmented brain; (b) sample coronal slice; (c) sample sagittal slice; (d) sample horizontal slice. In all panels, olfactory bulbs are shaded brown, cerebral hemispheres in purple, and posterior brain in green. The gray areas in panels (b–d) are non‐brain tissues. All images generated by 3D Slicer (v. 5.2.2).

We also examined the volumes of the subregions as a proportion of overall brain size. We found an interaction between weight and sex in both the cerebral hemispheres and the PB (CH: *F*(1,11) = 7.08, *p* = 0.022; PB: *F*(1,11) = 7.99, *p* = 0.017), but these interactions were in opposite directions: as a proportion of total brain volume, larger male snakes tended to have larger cerebral hemispheres, while the opposite pattern occurred in females (Figure 2a). In the PB, larger females tended to have larger volumes, whereas larger males tended to have less volume (Figure 2b). For both these brain regions, there were no main effects of weight (CH: *F*(1,11) = 3.49, *p* = 0.089; PB: *F*(1,11) = 0.27, *p* = 0.614), or sex (CH: *F*(1,11) = 0.06, *p* = 0.806; PB: *F*(1,11) = 0.06, *p* = 0.819). For the OBs, we did not find an interaction, but larger snakes had disproportionately larger OBs (Figure 2c; *F*(1,13) = 6.2, *p* = 0.027). In summary, enrichment tended to increase the brain volume of the snakes, especially in posterior regions of the brain. In addition, as female snakes grew, they tended to invest more volume into PB areas and their OBs. In contrast, as males grew, they invested more volume in the cerebral hemispheres and OBs. There was no effect of housing condition on the relative volume of any brain region.

## 4 Discussion

We investigated the effects of naturalistic EE on brain volume in juvenile western hognose snakes. We found that exposure to physical EE during the first year of life significantly increased total brain volume, particularly in PB regions (midbrain and hindbrain). Our findings add to growing evidence that reptiles respond to EE in a similar manner to other well‐studied animal models (Cummins et al. 1973; Scotto Lomassese et al. 2000; Näslund et al. 2012), in contrast to the common misconception that reptiles have a limited behavioral repertoire and are tolerant of minimalistic housing conditions (Warwick 1990; Case et al. 2005). As undisturbed natural environments are likely to be more complex and challenging than captive conditions, it may be more accurate to state that standard reptile housing conditions stunt brain growth and have negative effects on cognition and welfare. This is because captive environments are generally representative of what humans expect animals need, often leading to “controlled deprivation” (Burghardt 1996), in which captive environments lack many components necessary for healthy growth (Mendyk et al. 2023; Mendyk 2018).

The EE that we implemented took the form of naturalistic stimuli (substrate, climbing vines, large water dishes) that allowed snakes to engage in behaviors like climbing, burrowing, and swimming, thus encouraging various forms of locomotion. The enriched enclosures were also larger, providing more space for locomotion (Figure 1). Several studies observing the effects of enclosure size on reptiles have found that more space is generally positive. Corn snakes that were kept in enclosures large enough to completely stretch out were found to be more active and showed a significant preference for larger enclosures when allowed to actively choose (Hoehfurtner et al. 2021). Eastern blue‐tongued lizards (*Tiliqua scinoides*) utilized extra enclosure space when provided by engaging in more activity throughout the enrichment duration, encouraging healthy weight management (Philips et al. 2011). Various species of turtles also display an increase in the diversity of behaviors when exposed to EE in the form of larger space and more stimuli (Turner et al. 2022). The increased level of exercise available to snakes in the enriched condition may have been partially responsible for their larger brain volume, consistent with findings in other taxa (Cummins et al. 1973; Scotto Lomassese et al. 2000; Fong et al. 2019; Näslund et al. 2012).

There is very little literature on the anatomical effects of EE in reptiles. Juvenile male side‐blotched lizards displayed increased neurogenesis in the medial cortex when exposed to larger enclosures for 5 months, but the effect was confined to territorial males; no overall differences in brain volume were found (LaDage et al. 2013). This contrasts with research demonstrating larger overall brain volumes in wild territorial male side‐blotched lizards compared to non‐territorial males (LaDage et al. 2009). LaDage et al. (2013) explained these differences by suggesting that their EE lacked the complexity of the lizards’ natural environment. In line with this suggestion, our results indicate that the size and complexity of the environment are important for effects on brain volume in reptiles. Based on our findings, it is hard to discern whether this difference in brain size is a result of increased space or increased diversity of enrichment stimuli. Future research should determine the exact combination of enriching factors required for brain volume increases as well as how long animals need to spend under these conditions and whether there are critical periods for the effects.

The overall increase in brain volume we observed in enriched hognose snakes occurred primarily in the PB, made up of both the midbrain and the hindbrain and containing the optic tectum (midbrain), medulla oblongata, and cerebellum (hindbrain). Similar results have been reported in Atlantic salmon (Näslund et al. 2012). The snake cerebellum has been suggested to be extremely sensitive to locomotory behaviors, and its shape may depend on the types of locomotion snakes engage in (Macri et al. 2023). The significant increase in the size of the PB in our enriched snakes may be partly explained by increased demand on their cerebellums. In reptiles, the optic tectum is involved in visual and somatosensory processing (Catania et al. 2010), and the medulla oblongata in sensory and motor processing (Senn et al. 1970). As the enrichment we provided was both visually and tactilely more complex than the unenriched cages, it is possible that increased sensory complexity drove some of the increases in the size of these sensory‐motor regions.

In addition to EE‐driven differences in brain size, we also observed differences between sexes. As they grew larger, female juvenile snakes displayed a larger PB region while males displayed larger cerebral hemispheres, regardless of housing treatment. We note that, though our snakes were all approximately the same age, weight is a more important factor in determining maturity in snakes (Shine and Charnov 1992; Feldman and Meiri 2012). Sexual dimorphism in brain sizes is not uncommon and is evident in mammals, birds, and fish (Toli et al. 2017; Gittleman 1994; Garamszegi et al. 2005). In general, male and female snakes face similar environmental challenges, but they differ in breeding strategies and requirements, which may explain the differences we observed (Garamszegi et al. 2005).     

In conclusion, we found that physical enrichment increased overall brain size in hognose snakes—as it does in mammals, birds, and fish—mostly in PB regions. We also observed sex‐specific investments in different subregions of the brain. Our results, combined with earlier work on preference for enrichment (Nagabaskaran et al. 2022; Hoehfurtner et al. 2021) and its cognitive benefits (Nagabaskaran et al. 2021; Nagabaskaran 2020) in snakes, strongly suggest that snakes (and probably other reptiles) react to the complexity and diversity of behavioral opportunities in their environments in much the same way that mammals and birds do. We should therefore consider their needs in captivity in the same way as we do members of those other taxa.

## Conflicts of Interest

The authors declare no conflicts of interest.

### Peer Review

The peer review history for this article is available at https://publons.com/publon/10.1002/cne.70043.

## Data Availability

All data analyzed in this work are available in our OSF repository, at: https://osf.io/ph2bf/?view_only = 586213b7baea43c9811a0ae307aa7e0c [This repository is public].
